# Co- and Ni-Doped TiO_2_ Nanoparticles Supported on Zeolite Y with Photocatalytic Properties

**DOI:** 10.3390/nano13152200

**Published:** 2023-07-28

**Authors:** Gabriela Petcu, Florica Papa, Irina Atkinson, Adriana Baran, Nicoleta G. Apostol, Simona Petrescu, Lionel Richaudeau, Jean-Luc Blin, Viorica Parvulescu

**Affiliations:** 1 Institute of Physical Chemistry “Ilie Murgulescu”, Romanian Academy, 060021 Bucharest, Romania; gpetcu@icf.ro (G.P.); floricapapa@gmail.com (F.P.); iatkinson@icf.ro (I.A.); adibaran@gmail.com (A.B.); simon_pet@yahoo.com (S.P.); vpirvulescu@icf.ro (V.P.); 2 National Institute of Materials Physics, Atomiștilor 405A, 077125 Magurele, Romania; nicoleta.apostol@infim.ro; 3 Faculty of Sciences and Technology, University of Lorraine, CNRS, L2CM, F-54000 Nancy, France; lionel.richaudeau@univ-lorraine.fr

**Keywords:** Co-Ti/zeolite Y, Ni-Ti/zeolite Y, hierarchical zeolite Y, photodegradation, amoxicillin, mechanism

## Abstract

Zeolite Y samples with microporous and hierarchical structures containing Ti–Ni and Ti–Co oxides were obtained as active photocatalysts. Different Ti amounts (5, 10% TiO_2_) were supported, followed by the loading of Ni or Co oxides (5%). X-ray diffraction evidenced the presence of TiO_2_ as an anatase. N_2_ adsorption–desorption results showed type IV isotherms for hierarchical zeolite Y samples, and a combination of type IV and I isotherms for zeolite Y samples. UV–Vis diffuse reflectance spectra showed a shift in the absorption band to visible with increasing Ti loading and especially after Co and Ni addition. A significant effect of the support was evidenced for Ti and its interaction with Co/Ni species. The zeolite Y support stabilized Ti in the 4+ oxidation state while hierarchical zeolite Y support favored the formation of Ti^3+^ species, Ni^0^ and Ni^2+^ and the oxidation of Co to 3+ oxidation state. Photocatalytic activity, under UV and visible light irradiation, was evaluated by the degradation of amoxicillin, used as a model test. The photocatalytic mechanism was investigated using ethanol, p-benzoquinone and KI as ·OH and ·O_2_^−^ radicals and hole (h^+^) scavengers. The best results were obtained for the immobilized Ni-Ti species on the hierarchical zeolite Y support.

## 1. Introduction

The use of zeolite-based materials as adsorbents and photocatalysts for the extraction of heavy metals and the degradation of a wide variety of dyes, pharmaceutical compounds like antibiotics, and other organic pollutants has been broadly discussed [1,2,3,4], highlighting the advantage of their use in the treatment of contaminated water. Zeolites offer a high surface area, nanoporous structure, strong acidity, high ion exchange ability and hydrothermal stability for the synthesis of highly efficient photocatalysts. Unfortunately, the micropores from zeolites and active sites from the surface are less accessible to organic pollutants due to the limitations of mass transport. Furthermore, materials with a mesoporous structure are widely used as supports in the synthesis of photocatalysts because of their highly ordered mesoporous channels and large surface areas [5,6,7]. The solution to keep the properties of zeolites, along with the advantage of mass transfer through mesopores, seems to be hierarchical zeolites. These materials have significant advantages in reactions involving large molecules by coupling the native micropores with an intracrystalline mesoporous network. Higher adsorption capacity and degradation efficiency of amoxicillin were obtained for Au–Ti [7] and Fe–Ti [8] bimetallic photocatalysts supported on hierarchical zeolite Y photocatalysts. All zeolite supports are not photoactive, so they were activated by the incorporation of semiconductors and metal nanoparticles. The preparation of nanocomposite photocatalysts by immobilization of nanosized titanium dioxide into zeolite is a very promising solution to remove organic and inorganic pollutants from water [4,9,10,11]. The utilization of zeolite as a carrier for titanium dioxide led to smaller particle sizes of this oxide. Also, the photocatalytic activity of the TiO_2_–zeolite system increased compared to that of TiO_2_. However, TiO_2_ is a wide-band-gap semiconductor (~3.2 eV) and has a high recombination rate for electron–hole pairs with limited practical utility [12,13]. Recently, there has been increased interest in the synthesis of nanocomposite materials as photocatalysts that are more efficient and active under visible light irradiation by doping TiO_2_ with noble metals [7,14,15] and transition metal semiconductors [8,16,17,18,19]. Increasing catalytic activity with transition metals is an alternative to highly expensive noble metals. Transition metal cations have been found to be dopants and cocatalysts in photocatalytic reactions. Among different transition metal ions, Ni and Co have attracted attention for the development of visible-light-active TiO_2_ materials used in the photocatalytic degradation of organic pollutants [1,20,21,22,23,24,25,26]. At the interfacial contact between an n-type TiO_2_ semiconductor and a p semiconductor as nickel and cobalt oxides, a p–n heterojunction is established, which represents an effective way to hinder electron–hole pair recombination after light absorption and prolongs the charge carrier lifetime [27,28,29]. Theoretically, the p–n junction generates an internal electric field at the interface, which causes, at equilibrium, a negative charge in the p-type semiconductor region while the TiO_2_ region has a positive charge [27]. Under illumination, the photogenerated electron–hole pairs are separated by the internal electric field. Thus, the electrons move in the positive field and the holes in the negative field. Their separation leads to a significant increase in photocatalytic activity.

Cobalt oxide was reported as a good photocatalyst for pollutant degradation [5]. Bahnemann et al. [22] highlighted the effect of the synthesis method on the photocatalytic activity of TiO_2_ doped with cobalt. It was also shown that the photocatalytic activity under visible light irradiation largely depends on the valence state of the Co ions in the dopant and its concentration rather than the specific surface area of the support and the crystallinity of the anatase [5,21]. Also, in the case of TiO_2_ doping with NiO, it was evidenced [28] that the synergistic interactions of heterojunctions and the design, synthesis method and structure influenced the photocatalytic performances. PL spectra [30] showed that a small amount of Ni on TiO_2_ enhances the recombination of photogenerated electrons and holes, and Ni excess decreases the reaction rate due to the coverage effect of nickel species on the active site from the titanium dioxide surface.

While most works have focused on modifying TiO_2_-based photocatalysts in order of their increasing efficiency in the degradation of pollutants under sunlight or visible light irradiation, a limited number had as their objective the activation of zeolite-type supports and especially hierarchical zeolites. Also, a comparative study of the effect of Co and Ni doping on the activity of TiO_2_ immobilized on a zeolite support with a micro-mesoporous structure has not been carried out. The novelty also consists in evaluation with scavengers of the photocatalytic reaction mechanism.

In this work, we studied zeolite Y samples containing Ti–Ni and Ti–Co mixed oxides as photocatalysts for the degradation of amoxicillin in water. The zeolite Y supports, with microporous and hierarchical structures, were synthesized and impregnated, as active species, with different Ti amounts (5%, 10% TiO_2_), followed by loading with Ni or Co species (5% NiO, CoO). The effects of supports, TiO_2_ loading and type of the second immobilized metal (Ni or Co) on the formation of reactive species, photocatalytic performances and mechanisms were studied.

## 2. Materials and Methods

### 2.1. Materials

Sodium silicate solution (Na_2_O(SiO_2_)_x_·xH_2_O, reagent grade), sodium aluminate (NaAlO_2_), sodium hydroxide (NaOH, ≥98%), 1-propanol (CH_₃_CH_₂_CH_₂_OH, ACS reagent, ≥99.5%), tetradecyltrimethylammonium bromide (TTAB) (CH_3_(CH_2_)_13_N(Br)(CH_3_)_3_, for synthesis) and amoxicillin (C_16_H_19_N_3_O_5_S, 95.0–102.0% anhydrous basis), used for the photocatalytic reactions, and terephthalic acid (98%) were purchased from Sigma-Aldrich (Burlington, MA, USA). Titanium (IV) n-butoxide (Ti(OCH_2_CH_2_CH_2_CH_3_)_4_, reagent grade, 97%) was from ACROS Organics (Geel, Belgium). Nickel (II) nitrate ((Ni(NO_3_)_2_·6H_2_O) and cobalt (II) nitrate ((Co(NO_3_)_2_·6H_2_O) were from Merck (Darmstadt, Germany). Potassium iodide (KI) ethanol (C_2_H_5_OH) and p-benzoquinone (C_6_H_4_O_2_), used as scavengers, were purchased from Merck.

### 2.2. Photocatalysts Preparation

Hierarchical zeolite Y (noted hY) was obtained by a seed-assisted method, previously reported [16], in the presence of TTAB as a mesoporous structure-directing agent. After 24 h of aging, the synthesis mixture with the molar ratio 0.66Na_2_O:0.21Al_2_O_3_:SiO_2_:0.02TTAB:19.1H_2_O was hydrothermally treated for 6 h at 100 °C. Zeolite Y having only a microporous structure (noted Y) was obtained in a similar way, but in the absence of a surfactant. The precipitates obtained after the hydrothermal treatment were filtered, washed with deionized water until pH = 9, dried at 60 °C and calcined at 600 °C for 6 h.

Modification of zeolite Y and hierarchical zeolite Y with titanium dioxide was carried out by impregnation with an alcoholic solution of titanium butoxide. In the case of zeolite Y, the concentration of the immobilized TiO_2_ was 5%, while for the hierarchical zeolite hY, the percentage of TiO_2_ was varied (2, 5 and 10%). After impregnation, the samples were dried at room temperature for 24 h and then at 80 °C for 12 h. Calcination was carried out in air for 6 h at 600 °C with a heating rate of 2 °C/min. Further, these materials were impregnated with an aqueous solution of Co(NO_3_)_2_ or Ni(NO_3_)_2_, prepared so that the mass percentage of metal oxide in the final powders was 5%. After impregnation, the samples were dried at room temperature for 24 h and then at 60 °C for 8 h. Calcination was carried out in air for 6 h at 450 °C with a heating rate of 2 °C/min. The obtained materials were noted as hYT2C, hYT5C, YT5C and hYT10C in the case of Ti and Co oxide modification and as hYT2N, hYT5N, YT5N and hYT10N for Ti- and Ni-oxide-modified materials.

### 2.3. Photocatalysts Characterization

X-ray diffraction (XRD) patterns were recorded using a Rigaku Ultima IV diffractometer (Rigaku Corp., Tokyo, Japan) with Cu Kα, λ = 0.15406 nm. Phase analysis was performed using Rigaku PDXL software with the Whole Powder Pattern Fitting (WPPF) module, connected to the database ICDD-PDF-2.

Textural properties were evaluated using the TRISTAR 3000 sorptometer (Micromeritics, Merignac, France). Before measurements, the samples were outgassed under vacuum (pressure = 0.13 mBar) at 25 °C for 16 h.

Scanning electron microscopy (SEM) with EDX, FEI Quanta 3D FEG, was used to analyze the morphology and composition of the samples.

UV–Vis diffuse reflectance spectra of the powders were recorded in the 250–900 nm wavelength range using a JASCO V570 spectrophotometer (Tokyo, Japan).

Hydrogen temperature-programmed reduction (H_2_-TPR) experiments were performed using a ChemBET 3000-Quantachrome instrument (USA) equipped with a thermal conductivity detector (TCD). In total, 50 mg of photocatalyst was used, with a continuous flow of 5 vol% H_2_ in Ar (70 mL/min). The heating rate was 10 °C/min, up to 850 °C. In order to remove the water vapor and ensure optimal stability of the TCD (thermal conductivity detector), a silica gel column was inserted.

The analysis of the surface of the samples was investigated by X-ray photoelectron spectroscopy (XPS). XPS spectra were obtained in an AXIS Ultra DLD (Kratos Surface Analysis, Manchester, UK) setup, using Al Kα1 (1486.74 eV) radiation produced by a monochromatized X-ray source at an operating power of 192 W, and the high-resolution core-level spectra were recorded using the hybrid lens mode, 40 eV pass energy, slot aperture. The binding energy scale was calibrated to the C 1s standard value of 284.6 eV and the spectra of interest were deconvoluted using Voigt profiles, based on the methods described in Ref. [31].

Raman scattering spectra were collected on a Jobin-Yvon T64000 spectrometer equipped with an optical microscope in confocal mode (Horiba Jobin-Yvon, Palaiseau, France).

### 2.4. Photocatalytic Properties

The ability of the samples to generate, under irradiation, hydroxyl radicals in aqueous solution was evaluated with terephthalic acid. The fluorescence of 2-hydroxyterephthalic acid (TAOH) resulting from the interaction of terephthalic acid (TA, 5 × 10^−4^ M TA prepared using an aqueous solution of NaOH 2 × 10^−3^ M) with hydroxyl radicals generated under irradiation by the samples (λ_exc_ = 312 nm) was recorded using a FluoroMax 4P spectrofluorometer (Horiba Jobin Yvon, Northampton, UK).

The photocatalytic experiments were carried out in quartz microreactors (inner diameter of 21.5 mm and height of 37.2 mm), thermostated conditions (30 °C) and under stirring. Firstly, 10 mL of aqueous solution of amoxicillin (30 mg/L) and 20 mg of the photocatalyst were stirred for 30 min in darkness to allow AMX adsorption on the photocatalyst surface. Irradiation was then carried out for 5 h, using 2 × 60 W halogen lamps with 1.53 × 10^15^ photons/s/cm^2^ (UV) and 6.33 × 10^15^ photons/s/cm^2^ (visible). After 1, 3 and 5 h of irradiation, 3 mL was taken from the reaction mixture, the photocatalyst was separated using a Millipore syringe filter of 0.45 μm and the solution was spectrophotometrically measured using a JASCO V570 UV–Vis spectrophotometer (λ = 230 nm). AMX degradation was calculated using the solution’s phase concentration C_t_ (mg/L) at moment t and the initial concentration C_0_ (mg/L) at t = 0. The recycling studies were carried out under UV irradiation in 3 successive repetitions. The reaction time was 5 h. After each use, the photocatalyst was recovered by centrifugation, washed with distilled water and dried for 10 h at 100 °C.

For ROS scavenging tests, the experimental procedure was similar to the photocatalytic test. To each reaction system, 0.1 mmol of scavenger was added.

## 3. Results

### 3.1. Characterization of the Samples

#### 3.1.1. X-ray Diffraction

**Figure 1:**
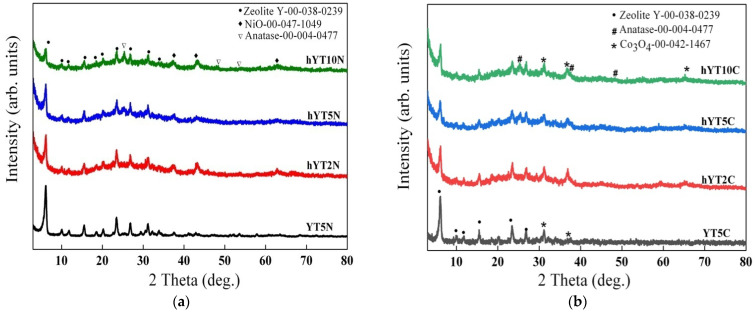
X-ray diffractograms of (**a**) Ni-Ti/zeolite Y and (**b**) Co-Ti/zeolite Y samples (Y—zeolite Y with microporous structure; hY—zeolite Y with hierarchical structure).

The wide-angle XRD patterns of the synthesized samples are illustrated in Figure 1a,b. For all the samples, the diffraction pattern of zeolite Y (ICDD 00-038-0239), used as a support to obtain the photocatalysts, was evidenced. Furthermore, for all the prepared samples, a diffraction peak located at 25.2° was noticed, assigned to anatase [32], the intensity of which increases with TiO_2_ concentration (from 2% to 10%), most likely as a result of the size variation in the supported TiO_2_ nanoparticles. The X-ray diffractograms presented in Figure 1a confirm the presence of nickel as NiO species in the samples after impregnation, by the appearance of characteristic diffraction lines at 37.3°, 43.3° and 62.9° [33]. In the case of modification with cobalt, the XRD results suggest the formation of Co_3_O_4_ (Figure 1b), associated with the presence of some discrete diffraction peaks located at values of 2θ = 31.2°, 36.7° and 65.7° (ICDD 00-042-1467).

#### 3.1.2. N_2_-Sorption

**Figure 2:**
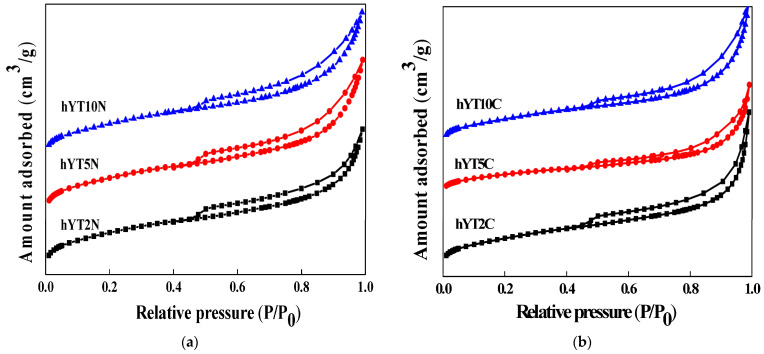
The nitrogen adsorption–desorption isotherms of the zeolitic samples modified with (**a**) Ti–Ni oxides and (**b**) Ti–Co oxides.

The nitrogen adsorption–desorption isotherms recorded for the materials obtained by impregnation with titania and nickel or cobalt oxide are shown in Figure 2.

No change in the isotherm type, which is characteristic of the hY support (type IV), was evidenced after double impregnation processes (Figure 2). The specific surface area and pore volume values are shown in Table 1.

A significant decrease in the specific surface after double impregnation with metal oxides was revealed, suggesting the blocking of the pores by the distribution of active species inside the micro- and mesopores.

#### 3.1.3. Scanning Electron Microscopy (SEM)

**Figure 3:**
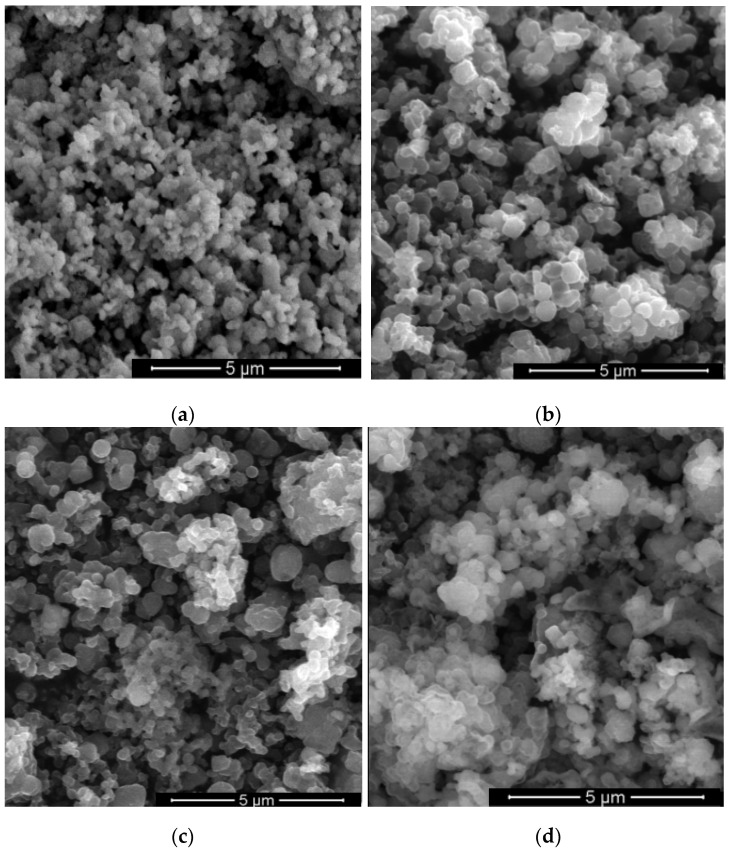

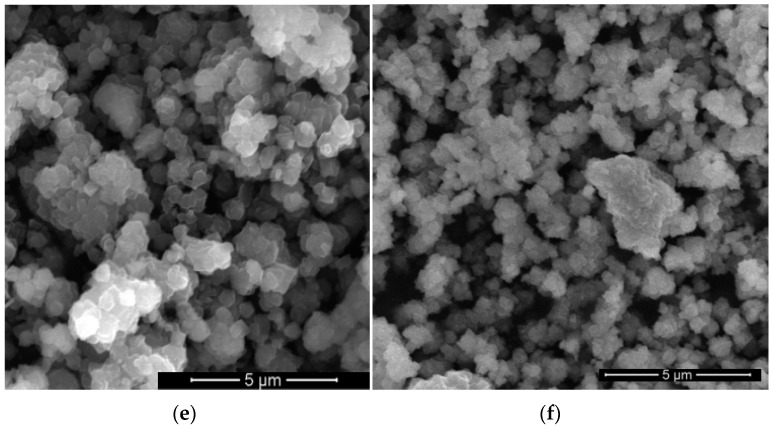
SEM images of (**a**) hYT2N, (**b**) hYT10N, (**c**) hYT10C, (**d**) hYT5C, (**e**) YT5C and (**f**) zeolite Y samples.

The morphology of the zeolite Y before and after its impregnation with Ti and Co or Ni precursors was evaluated by scanning electron microscopy (Figure 3). No change in morphology was observed after the impregnation of supports. Thus, the preservation of octahedral morphology with a smooth surface, specific to zeolite Y, was evidenced.

**Figure 4:**
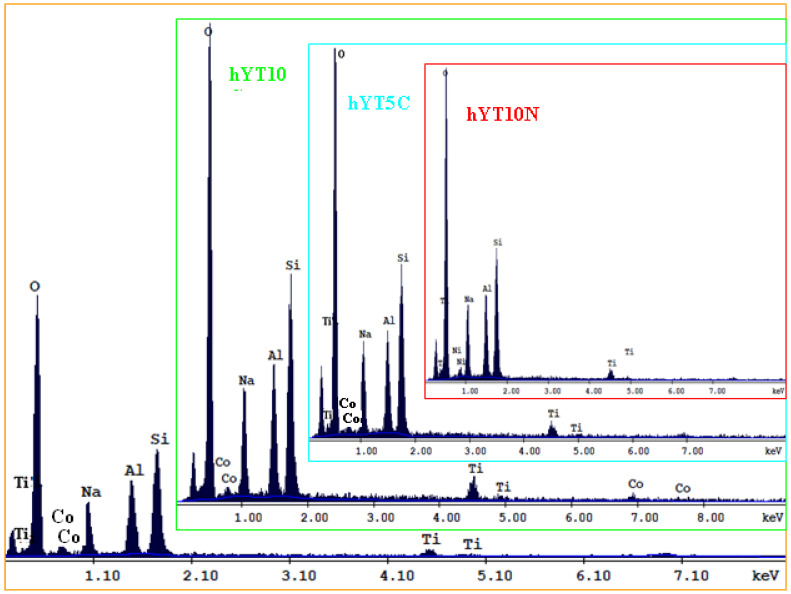
EDX spectrum of Co–Ti- and Ni–Ti-supported samples.

The composition of the samples was evaluated by EDX analysis. The elemental analysis (Figure 4) shows the presence of immobilized species Ti, Co or Ti, Ni (Figure 4) and a higher content of elements (O, Si, Al, Na) from the composition of the zeolite supports.

#### 3.1.4. UV–Vis Absorption Spectroscopy

**Figure 5:**
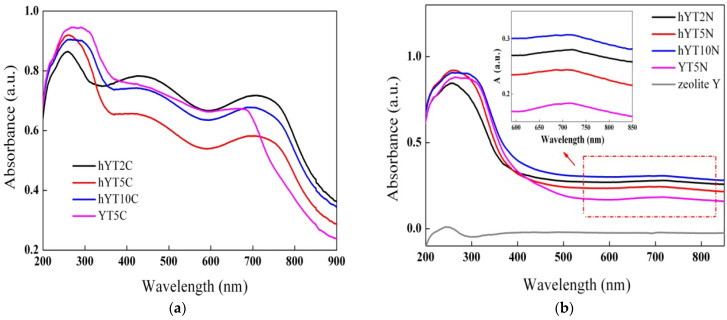
UV–Vis absorption spectra of (**a**) Ti-Co-modified samples and (**b**) Ti-Ni-modified samples.

The optical properties of the samples were investigated by UV–Vis absorption spectroscopy, and the results are shown in Figure 5. It is observed that the zeolitic support does not absorb light in the UV–Vis range; therefore, the optical properties are only due to the supported oxide species. Figure 5 shows the presence of a broad absorption band for all the samples in the UV range, with a maximum peak located at ~260 nm, assigned to ligand-to-metal charge transfer of the highly dispersed octahedrally coordinated titanium oxide species. By increasing the TiO_2_ amount from 2% to 10%, a broadening of the UV absorption band towards wavelengths over 300 nm can be noticed, most likely due to the growth of the anatase particle size [34,35].

Furthermore, in the case of Co-modified samples (Figure 5a), the presence of two absorption bands in the visible domain can be noticed, specific to Co_3_O_4_ species, with maxima located at ~440 nm and ~710 nm [36]. These are assigned to O^2−^ → Co^2+^ and O^2−^ → Co^3+^ charge transfers and also to 1A_1g_ → 1T_2g_ electron transfer given by the octahedral Co(III) species. The hypsochromic shift in the characteristic absorption band of unsupported Co_3_O_4_ (located at 470 nm, as reported in the literature) indicates the obtaining of smaller-sized Co_3_O_4_ species by dispersion on the zeolitic Y support [37]. The same behavior was observed for the Ni-modified samples, which exhibited a broad absorption band with the maximum located at ~715 nm, assigned to NiO species (Figure 5b), and not at 725 nm, as was reported for the unsupported NiO [38].

The recorded UV–Vis absorption spectra indicate that the synthesized materials are active in both UV and visible light irradiation. Obtaining materials with visible light absorption capacity by modifying the zeolitic supports with Co or Ni oxide is an extremely important aspect in the current context, where the maximization of efficiency is desired but with minimal energy consumption [21], and was also supported by the band gap values calculated using the Kubelka–Munk function (Table 1).

#### 3.1.5. H_2_-TPR Measurements

The reduction behavior of the synthesized photocatalysts was studied by thermoreduction measurements (H_2_-TPR) to investigate the chemical state of the metal as well as to obtain information about the reducibility of the species on the photocatalyst surface and in the bulk. A strong interaction of the immobilized metal species with hierarchical zeolite Y can lead to changes in chemical status. A weak interaction with the support favors the reduction of metal oxides at lower temperatures, while a strong interaction with the support forms species that reduce at higher temperatures. The amount of H_2_ (µmol) consumed per gram of photocatalyst is indicated in Table 2.

**Figure 6:**
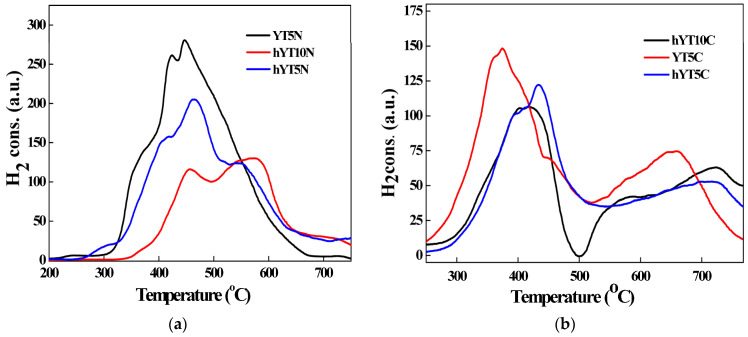
H_2_–TPR profiles of the samples with (**a**) Ti–Ni oxides and (**b**) Ti–Co oxides.

The TPR profiles recorded for the samples are illustrated in Figure 6a,b. In the case of materials modified with Ti and Ni oxides, the TPR profiles (Figure 6a) show several signals that can be divided as follows: peaks occurring at temperatures below 500 °C are attributed to various NiO species mainly formed on the outer surface of the zeolite, peaks occurring in the range of 500–650 °C are due to Ni ions present inside the zeolitic channels and peaks located at temperatures higher than 700 °C are attributed to Ni species incorporated in the zeolitic network, according to the literature [39]. For the YT5N sample, no reduction peak in the temperature range of 500–650 °C was observed, which means that the microporous structure of the support did not allow the access of Ni ions inside the zeolitic channels with smaller dimensions than in the case of hierarchical zeolite. There is also a shift in the signals recorded for the hYT10N sample to higher values of reduction temperatures. This indicates stronger interactions between Ni oxide and supported titanium dioxide [40], most likely due to the high concentration of TiO_2_ (10%), which ensures a higher probability of interaction with nickel oxide. The high degree of interaction between NiO and TiO_2_ in this sample is also supported by the absence of reduction peaks of bulk NiO, which usually occur in the temperature range between 280 and 300 °C. In the case of a moderate interaction of NiO with TiO_2_, its reduction takes place at about 400 °C, while in the case of stronger interactions with titanium dioxide, the reduction temperature of NiO increases to 530 °C [41,42]. The presence of reduction peaks up to 500 °C for all the samples modified with Ti–Ni mixed oxides indicates the formation of NiO species, predominantly on the outer surface of zeolitic materials [40] (both zeolite Y and hierarchical zeolite hY). The consumption of hydrogen from temperatures higher than 600 °C can be attributed to the reduction of titanium. The presence of metallic nickel particles on the zeolite subsurface favors the molecular dissociation of H_2_, and thus the reduction of Ti^4+^ to Ti^3+^ or Ti species with a lower valence state. The amount of hydrogen needed to reduce nickel is greater than the stoichiometric amount. This confirms the hypothesis that the reduction of small amounts of titanium also takes place.

Figure 6b shows the TPR profiles for samples containing Ti–Co with three different domains of reduction temperatures. The first peaks observed at a lower temperature (200 °C) correspond to the reduction to CoO on the surface of the support (I); the second and third peaks located at 370 and 536 °C (II and III) can be attributed to the reduction of Co^3+^ → Co^2+^ → Co^0^; the fourth peak at 636 °C can be attributed to the reduction of Co^2+^, which strongly interacts with the support [43,44,45,46,47,48]. TPR signals recorded at higher temperatures, between 600 and 700 °C, are due to the reduction of cobalt oxide species strongly bound to the support (surface Co–O–Al species reduced to Co metal), as reported in the case of Co_3_O_4_ on alumina support [48].

#### 3.1.6. XPS Analysis

**Figure 7:**
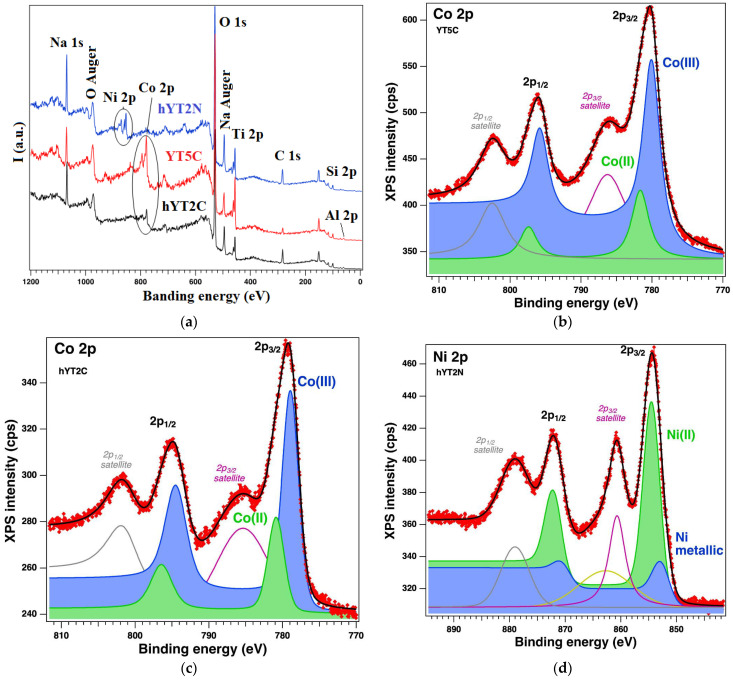

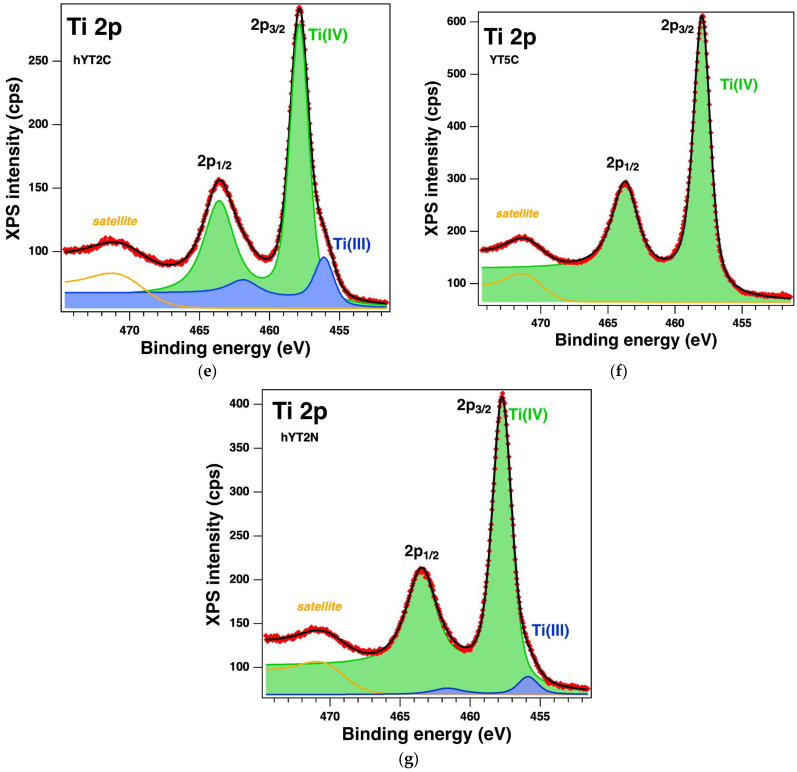
XPS spectra: (**a**) general survey for the samples hYT2N (blue line), YT5C (red line) and hYT2C (black line); (**b**,**c**) Co 2p core level for YT5C and hYT2C samples, respectively; (**d**) Ni 2p core level for hYT2N sample and (**e**–**g**) Ti 2p core level for hYT2C, YT5C and hYT2N samples, respectively. For all the core levels red symbols—raw data, black line—fit, blue and green solid colours—two main components (for (**f**) only one main component); the other coloured lines—components attributed to satellites.

The surface of the samples and the oxidation states were investigated by XPS, which revealed the existence of the following core levels: O 1s, Si 2p, Al 2p, Na 1s, C 1s, Co 2p, Ti 2p and Ni 2p. The spectra of interest are illustrated in Figure 7. Table 3 gives the relevant parameters obtained after the deconvolutions of the core levels of interest (Ti 2p, Co 2p and Ni 2p), i.e., binding energies, amplitudes and assignment, by consulting the existing database. The core levels Co 2p, Ni 2p and Ti 2p were analyzed (“deconvoluted”) using doublet Voigt profiles and singlets for the satellites. For the Co 2p doublet, we have four main peaks: between 770 and 790 eV, there are peaks of Co 2p_3/2_ and its satellite, and at higher binding energies are Co 2p_1/2_ and its satellite. The spin orbit splitting in this case is between 15.5 and 15.8 eV. The “deconvolutions” revealed the coexistence of Co 2^+^ and Co^3+^. The same algorithm was used for Ni 2p and Ti 2p according to the ref. [49,50]. These are all the peaks, doublets and their satellites.

Thus, it can be observed that for sample YT5C, based on zeolite Y support, no titanium reduction occurs and there are fewer oxidized Co species (Table 3). Instead, the formation of Ti(III) is observed in the case of samples supported on hierarchical zeolite Y. At the same time, several species of oxidized Co(III) are formed on this support, even if the percentage of TiO_2_ is lower. We consider that this is the result of different interactions of titanium species with the surface of zeolite Y and that of aluminosilicate with mesopores.

#### 3.1.7. Raman Spectroscopy

**Figure 8:**
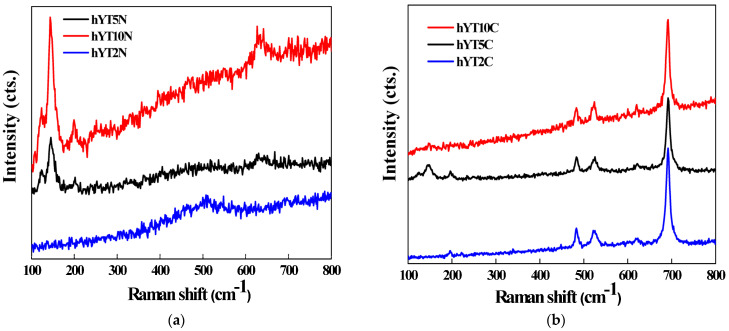
Raman spectra of (**a**) hYT2N, hYT5N, hYT10N and (**b**) hYT2C, hYT5C, hY110C samples.

Raman spectra of the synthesized samples are illustrated in Figure 8. For samples containing Ti–Ni (Figure 8a), Raman modes of segregated anatase are depicted at 144 (E_g_), 197 (E_g_) and 637 cm^−1^ (E_g_), while for the samples modified with Ti and Co oxides, more obvious are the peaks located at 192, 483, 527, 620 and 692 cm^−1^ (Figure 8b), which characterize the Raman active modes of Co_3_O_4_. The phonon symmetries of these Raman peaks are caused by lattice vibrations of the spinel structure, in which Co^2+^ and Co^3+^ cations are situated at tetrahedral and octahedral sites in the cubic lattice [36].

### 3.2. Photocatalytic Activity

#### 3.2.1. Photocatalytic Results

**Figure 9:**
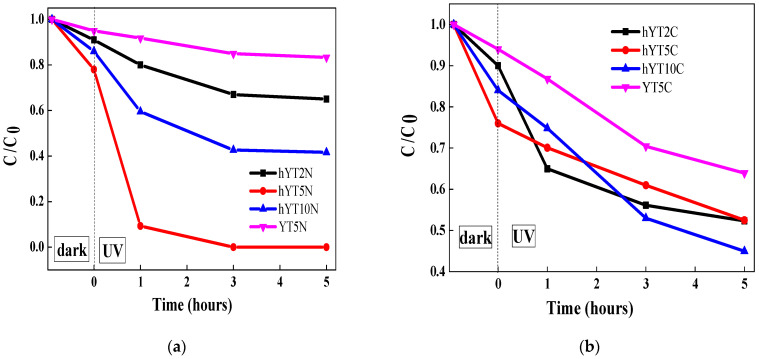

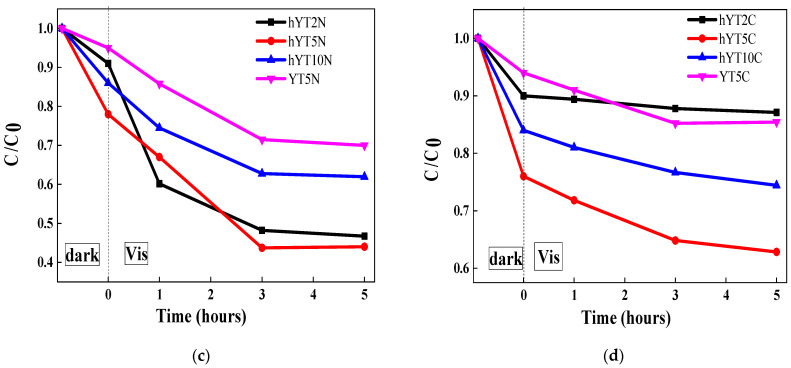
Photocatalytic degradation of amoxicillin using the synthesized materials under UV (**a**,**b**) and visible light (**c**,**d**) irradiation.

The synthesized materials were tested in the photocatalytic degradation of amoxicillin under UV and visible light irradiation. The results obtained are presented in Figure 9a–d. In the case of UV light irradiation (Figure 9a,b), a higher photocatalytic efficiency of materials based on hierarchical zeolite Y was observed. This behavior is due to its characteristic properties which contribute to a high and uniform dispersion of metal species and also improve the access of both light radiation and amoxicillin molecules to the metallic sites. Similar results were noticed under visible light irradiation (Figure 9c,d), highlighting that the hierarchical zeolite Y used as a support improves the photocatalytic performances of the resulting materials compared to the non-hierarchical zeolite Y.

In the first 30 min in the dark, the concentration of amoxicillin decreases as a result of its desorption on the surface of the photocatalyst. The adsorbed amount varies with the tested material. The highest adsorption capacity has the samples supported on hierarchical zeolite Y modified with TiO_2_. The effect of Co or Ni oxides is insignificant.

**Figure 10:**
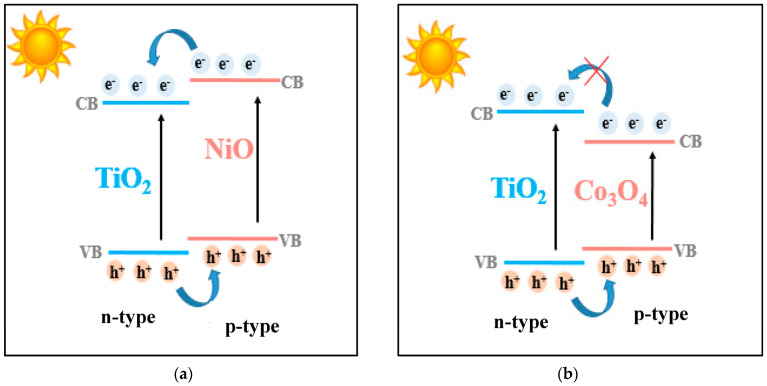
(**a**,**b**) Schematic representation of charge transfer in the p–n heterojunction of the synthesized materials.

It was pointed out that Ti–Ni-modified photocatalysts have better activity than those modified with Ti and Co oxides. This is explained by the efficient separation of photogenerated electron–hole pairs, as a synergistic effect of the internal electric field resulted in a typical p–n heterojunction and band alignment of NiO and TiO_2_ semiconductors, as can be seen in Figure 10a. In contrast, for the photocatalytic systems containing TiO_2_–Co_3_O_4_, the electron transfer across the electric field is thermodynamically hindered, because the conduction band of the Co_3_O_4_ semiconductor is lower than that of TiO_2_ (Figure 10b) [51].

**Figure 11:**
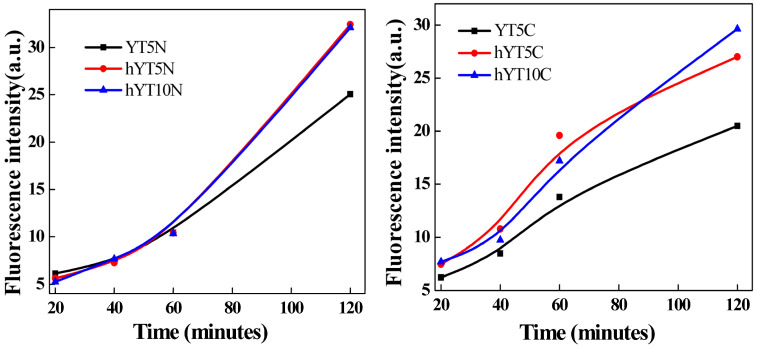
Fluorescence emission spectra of 2-hydroxyterephthalic acid that resulted after interaction between terephthalic acid and ·OH radicals.

Better separation of charge carriers in the case of Ti–Ni samples leads to a higher number of holes available to generate more hydroxyl radicals (·OH), species responsible for the degradation of amoxicillin in photocatalytic reactions [16]. The ability of these materials to generate hydroxyl radicals was also supported by the fluorescence results obtained in the case of terephthalic acid reaction (Figure 11). This method is based on the capacity of terephthalic acid (TA) to interact with ·OH radicals, which resulted in an aqueous solution during the photocatalytic experiment, leading to the formation of a photoluminescence compound, 2-hydroxyterephthalic acid (TAOH), which can be quantified.

**Figure 12:**
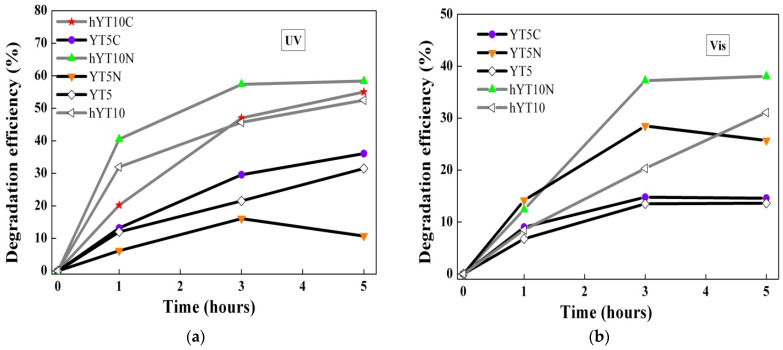
Effect of Co, Ni doping on photocatalytic activity under UV and Visible light irradiation.

An increase in the photocatalytic efficiency was noticed by doping with cobalt or nickel, especially under visible irradiation (Figure 12).

Figure 12 shows the effect of Co or Ni immobilization on the photocatalytic efficiency of the zeolite Y support modified with TiO_2_. In most reactions, a smaller or larger increase in activity is observed, except when the reaction is carried out in UV using the photocatalyst with nickel oxide immobilized on YT5. It is considered that nickel immobilization can inhibit, in UV, the activity of TiO_2_ supported on zeolite Y with lower values of pores and specific surface areas.

#### 3.2.2. Mechanism Investigations

**Figure 13:**
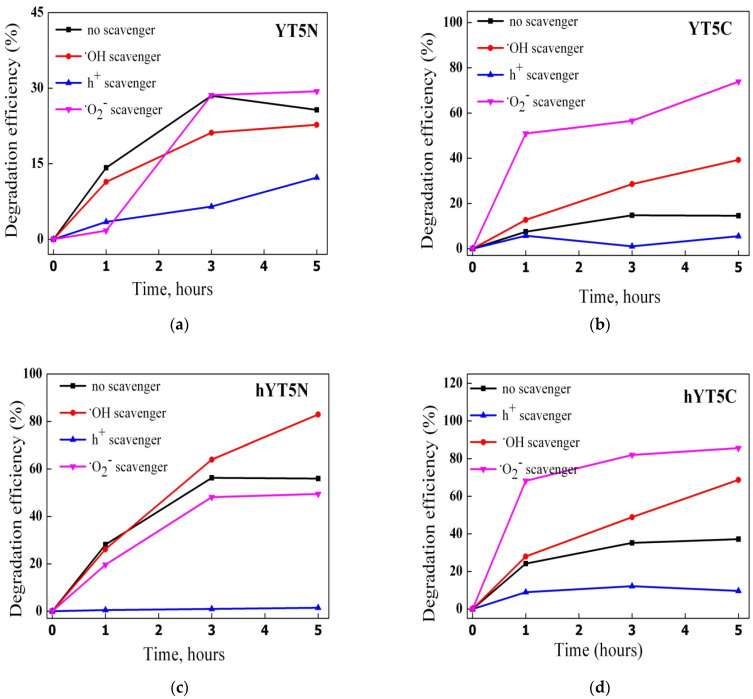

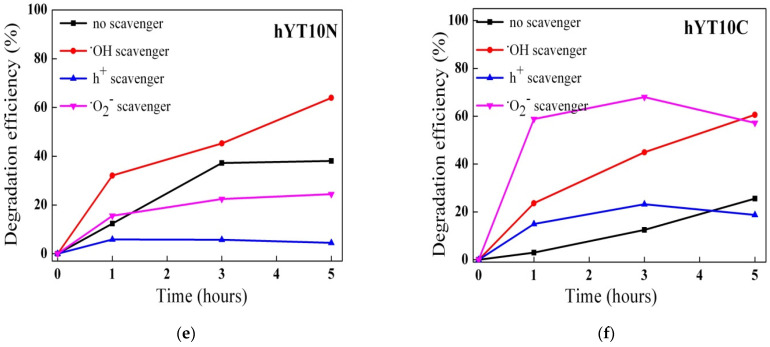
The effect of scavengers on the photocatalytic degradation of amoxicillin under visible light irradiation for (**a**) YT5N, (**b**) YT5C, (**c**) hYT5N, (**d**) hYT5C, (**e**) hYT10N and (**f**) hYT10C samples.

Generally, the main reactive species in the photocatalytic degradation of organic pollutants are hydroxyl (·OH), superoxide (·O_2_^−^) and hole (h^+^) radicals [37]. To evaluate the mechanism of amoxicillin degradation using the synthesized materials, photocatalytic experiments were carried out in the presence of scavengers able to selectively interact with the highly reactive oxygen species (ROS) from the reaction system. The results obtained from these experiments are shown in Figure 13.

A different contribution of reactive oxygen species to AMX degradation depending on the metal species used to modify the zeolitic support was noticed. Thus, in the case of the samples containing Ti and Ni oxides, the following order was observed: h^+^ > ·O_2_^−^ > ·OH. For the samples modified with Ti and Co oxides, the order was h^+^ > ·OH> ·O_2_^−^. The decrease in the degradation efficiency after KI addition suggests that holes are mainly responsible for AMX degradation. In fact, the holes have a dual role: they are directly involved in the degradation of amoxicillin molecules and in the formation of reactive hydroxyl species (·OH) through the oxidation of water.

Interestingly, by adding scavengers for ·O_2_^−^ and ·OH species, an increase in photocatalytic efficiencies was recorded. This means that the lack of an active species in the system is compensated by others with higher reactivity, depending on their oxidizing potential. For example, by capturing superoxide species, it is practically possible for amoxicillin molecules to be oxidized by hydroxyl radicals, known to be the most active of all reactive oxygen species [52], hence the increase in catalytic performance in the case of adding p-benzoquinone (p-BQ) to the system, as was obtained for the samples modified with titanium and cobalt oxides (Figure 13b,d,f).

**Figure 14:**
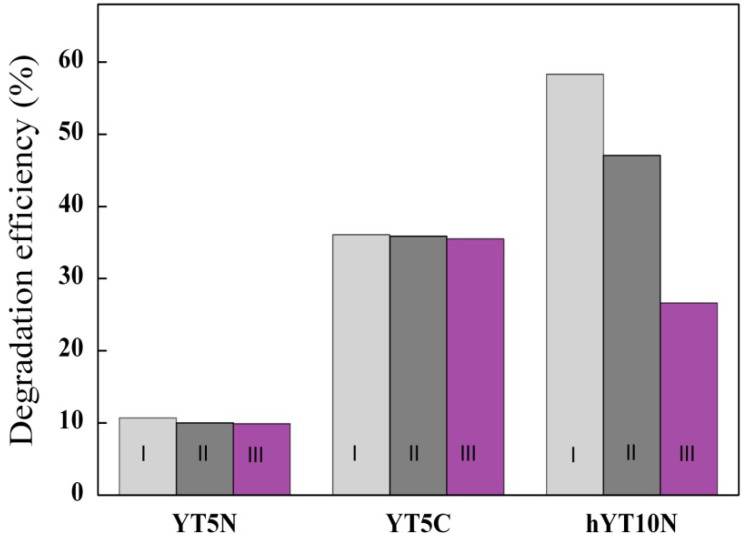
Performance of recycled YT5N, YT5C and hYT10N samples for AMX degradation under UV light irradiation (I—1st cycle, II—2nd cycle, III—3rd cycle).

The results of the recycling study (Figure 14) show high stability for the zeolite Y samples with a microporous structure.

A halving of the photocatalytic efficiency was observed for the hYT10N sample after three repeated testing cycles, probably due to a higher adsorption capacity of AMX and degradation products on hierarchical zeolite Y, leading to a blocking of the active sites [8].

## 4. Conclusions

New Co–Ti- or Ni–Ti-supported photocatalysts on zeolite Y with microporous and hierarchical structures were obtained, and their high activity under UV and visible light was evidenced by amoxicillin photodegradation. A typical crystalline structure of zeolite Y and the presence of mesopores were evidenced for all the obtained samples. A significant effect of the support was observed on Ti and Co/Ni species and their interaction. It was thus highlighted that the zeolite Y support stabilizes Ti in the 4+ oxidation state and reduces the oxidation of Co or Ni species. In the case of the hierarchical zeolite Y, reduced species of Ti(III) are formed on the surface, and the amount of the other oxidized metal species increases significantly in the case of nickel. The presence of Co and Ni species in 3+ and 2+ oxidation states was also confirmed by Raman spectroscopy and H_2_–TPR results. The mechanism of amoxicillin degradation under UV and visible light irradiation, investigated in the presence of scavengers, evidenced the effect of metal species interaction and zeolite support. The best results were obtained for the immobilized Ni–Ti species and hierarchical zeolite Y support.

## Figures and Tables

**Table 1:** The specific BET surface areas (S_BET_), pore volume (V_pore_) and band gap values of the synthesized photocatalysts.

Sample	hY	hYT2N	hYT5N	hYT10N	hYT2C	hYT5C	hYT10C
S_BET_ (m^2^/g)	810	100	98	91	99	50	89
V_pore_ (cm^3^/g)	0.2	0.08	0.08	0.08	0.1	0.06	0.09
E_g_ (eV)	-	2.18	2.93	2.88	1.55	2.51	2.05

**Table 2:** The amount of hydrogen consumed, calculated from the TPR profiles of the samples.

Sample	The Metallic Species	µmol H_2_/g
YT5C	Co^2+^, Co^3+^	1109
YT5N	Ni^2+^, Ni^0^	869
hYT10C	Co^2+^, Co^3+^, Ti^4+^, Ti^3+^	1151
hYT10N	Ni^2+^,Ni^0^	916
hYT5N	Ti^4+^,Ti^3+^	858
hYT5C	Co^3+^, Co^2+^	1090

**Table 3:** Binding energies (BE), amplitudes and assignment of the deconvolutions for the core levels of interest for the samples.

Sample	Element		BE (eV)	Ampl. (cps)	Assignment
hYT2C	Ti2p	C1	456.06	84.85	Ti(III)
		C2	458.15	408.71	Ti(IV)
		Ti(III)/Ti(IV)			0.21
	Co 2p	C1	778.92	480.6	Co(III)
		C2	780.88	214.7	Co(II)
		Co(II)/Co(III)			0.45
YT5C	Ti 2p	C1	458.07	242.65	Ti(IV)
	Co 2p	C1	780.04	1229.39	Co(III)
		C2	781.65	471.77	Co(II)
		Co(II)/Co(III)			0.38
hYT2N	Ti 2p	C2	456.04	77.38	Ti(III)
		C3	458.15	223.11	Ti(IV)
		Ti(III)/Ti(IV)			0.35
	Ni 2p	C1	852.75	124.61	Ni metallic
		C2	854.5	673.48	Ni(II)
		Ni(0)/Ni(II)			0.19

## Data Availability

The data presented in this study are available upon request from the corresponding author.

## References

[1] Zhao Z., Wang N., Zhang H., Shang R., Xing J., Zhang D., Li J. (2022). Fabrication of ZSM-5 zeolite supported TiO_2_-NiO heterojunction photocatalyst and research on its photocatalytic performance. J. Solid State Chem..

[2] Umejuru E.C., Mashifana T., Kandjou V., Amani-Beni M., Sadeghifar H., Fayazi M., Karimi-Maleh H., Sithole N.T. (2023). Application of zeolite based nanocomposites for wastewater remediation: Evaluating newer and environmentally benign approaches. Environ. Res..

[3] Samaniego-Benítez J.E., García-García A., Rivera-Manrique S.I., Ramírez-Aparicio J. (2023). Multiwalled carbon nanotubes/zeolite composite for dye degradation under sunlight. Mater. Today Commun..

[4] Hu G., Yang J., Duan X., Farnood R., Yang C., Yang J., Liu W., Liu Q. (2021). Recent developments and challenges in zeolite-based composite photocatalysts for environmental applications. Chem. Eng. J..

[5] Suraja P.V., Yaakob Z., Binitha N.N., Resmi M.R., Silija P.P. (2011). Photocatalytic degradation of dye pollutant over Ti and Co doped SBA-15: Comparison of activities under visible light. Chem. Eng. J..

[6] Huang C.H., Chang K.P., Ou H.D., Chiang Y.C., Chang E.E., Wang C.F. (2011). Characterization and application of Ti-containing mesoporous silica for dye removal with synergistic effect of coupled adsorption and photocatalytic oxidation. J. Hazard. Mater..

[7] Petcu G., Anghel E.M., Buixaderas E., Atkinson I., Somacescu S., Baran A., Culita D.C., Trica B., Bradu C., Ciobanu M. (2022). Au/Ti Synergistically Modified Supports Based on SiO_2_ with Different Pore Geometries and Architectures. Catalysts.

[8] Petcu G., Anghel E.M., Somacescu S., Preda S., Culita D.C., Mocanu S., Ciobanu M., Parvulescu V. (2020). Hierarchical Zeolite Y Containing Ti and Fe Oxides as Photocatalysts for Degradation of Amoxicillin. J. Nanosci. Nanotechnol..

[9] Diban N., Pacuła A., Kumakiri I., Barquín C., Rivero M.J., Urtiaga A., Ortiz I. (2021). TiO_2_–Zeolite Metal Composites for Photocatalytic Degradation of Organic Pollutants in Water. Catalysts.

[10] Joseph C.G., Sharain-Liew Y.L., Bono A., Teng L.Y. (2013). Photodegradation of Indigo Dye Using TiO_2_ and TiO_2_/Zeolite System. Asian J. Chem..

[11] Chatti R., Rayalu S.S., Dubey N., Labhsetwar N., Devotta S. (2007). Solar-based photoreduction of methyl orange using zeolite supported photocatalytic materials. Sol. Energy Mater. Sol. Cells.

[12] Das A., Mrinal K., Adak M.K., Mahata N., Biswas B. (2021). Wastewater treatment with the advent of TiO_2_ endowed photocatalysts and their reaction kinetics with scavenger effect. J. Mol. Liq..

[13] Chen D., Cheng Y., Zhou N., Paul Chen P., Wang Y., Kun Li K., Huo S., Cheng P., Peng P., Zhang R. (2020). Photocatalytic degradation of organic pollutants using TiO_2_-based photocatalysts: A review. J. Clean. Prod..

[14] Sescu A.M., Favier L., Lutic D., Soto-Donoso N., Ciobanu G., Harja M. (2021). TiO_2_ Doped with Noble Metals as an Efficient Solution for the Photodegradation of Hazardous Organic Water Pollutants at Ambient Conditions. Water.

[15] Goncearenco E., Morjan I.P., Dutu E., Scarisoreanu M., Fleaca C., Gavrila-Florescu L., Dumitrache F., Banici A.M., Teodorescu V.S., Anastasescu C. (2022). The effect of noble metal addition on the properties of oxide semiconductors nanoparticles. J. Solid State Chem..

[16] Krishnan A., Swarnalal A., Das D., Krishnan M., Saji V.S., Shibli S.M.A. (2023). A review on transition metal oxides based photocatalysts for degradation of synthetic organic pollutants. J. Environ. Sci..

[17] Amorós-Pérez A., Cano-Casanova L., Castillo-Deltell A., Lillo-Ródenas M.A., Román-Martínez M.C. (2019). TiO_2_ Modification with Transition Metallic Species (Cr, Co, Ni, and Cu) for Photocatalytic Abatement of Acetic Acid in Liquid Phase and Propene in Gas Phase. Materials.

[18] Suligoj A., Arcon I., Mazaj M., Drazic G., Arcon D., Cool P., Stangar U.L., Tusar N.N. (2018). Surface modified titanium dioxide using transition metals: Nickel as a winning transition metal for solar light photocatalysis. J. Mater. Chem. A.

[19] Filip M., Petcu G., Anghel E.M., Petrescu S., Trica B., Osiceanu P., Stanica N., Atkinson I., Munteanu C., Mureseanu M. (2021). FeTi-SBA-15 magnetic nanocomposites with photocatalytic properties. Catal. Today.

[20] Jo W.-K., Kumar S., Isaacs A.A., Lee A.F., Karthikeyan S. (2017). Cobalt promoted TiO_2_/GO for the photocatalytic degradation ofoxytetracycline and Congo Red. Appl. Catal. B Environ..

[21] Iwasaki M., Hara M., Kawada H., Taday H., Ito S. (2000). Cobalt Ion-Doped TiO_2_ Photocatalyst Response to Visible Light. J. Colloid Interface Sci..

[22] Akel S., Boughaled R., Dillert R., El Azzouzi M., Bahnemann D.W. (2020). UV/Vis Light Induced Degradation of Oxytetracycline Hydrochloride Mediated by Co-TiO_2_ Nanoparticles. Molecules.

[23] Li J., Liu S., He Y., Wang J. (2008). Adsorption and degradation of the cationic dyes over Co doped amorphous mesoporous titania–silica catalyst under UV and visible light irradiation. Microporous Mesoporous Mater..

[24] Najafabadi A.T., Taghipour F. (2012). Cobalt precursor role in the photocatalytic activity of the zeolite-supported TiO_2_-based photocatalysts under visible light: A promising tool toward zeolite-based core–shell photocatalysis. J. Photochem. Photobiol. A Chem..

[25] Song Z., Liu J., Hu Y., Li S., Zhang X., Ma L., Chen L., Zhang Q. (2023). Solvent-controlled selective photocatalytic oxidation of benzyl alcohol over Ni@C/TiO_2_. Catal. Commun..

[26] Lu X., Liu F., Dang Y., Li M., Ruan M., Wu M., Zhu C., Mani T., Suib S.L., Gao P.-X. (2022). Transition-metal doped titanate nanowire photocatalysts boosted by selective ion-exchange induced defect engineering. Appl. Surf. Sci..

[27] Daia G., Liua S., Lianga Y., Luo T. (2013). Synthesis and enhanced photoelectrocatalytic activity of p–n junction Co_3_O_4_/TiO_2_ nanotube arrays. Appl. Surf. Sci..

[28] Chen J., Wang M., Han J., Guo R. (2020). TiO_2_ nanosheet/NiO nanorod hierarchical nanostructures: P–n heterojunctions towards efficient photocatalysis. J. Colloid Interface Sci..

[29] Uddin T., Nicolas Y., Oliver C., Jaegermann W., Rockstroh N., Junge H., Toupance T. (2017). Band alignment investigations of heterostructure NiO/TiO_2_ nanomaterials used as efficient heterojunction earth-abundant metal oxide photocatalysts for hydrogen production. Phys. Chem. Chem. Phys..

[30] Tseng Y.-H., Huang B.-K. (2012). Photocatalytic Degradation of NO*x* Using Ni-Containing TiO_2_. Int. J. Photoenergy.

[31] Teodorescu C.M., Esteva J.M., Karnatak R.C., El Afif A. (1994). An approximation of the Voigt I profile for the fitting of experimental x-ray absorption data. Nucl. Instrum. Meth. Phys. Res. A.

[32] Shindo T., Koizumi N., Hatekeyama K., Ikeuchi T. (2011). Post-synthesis of TiO_2_ Dispersed Inside the Pore Channels of SBA-15 and its Photocatalytic Activity for the Degradation of Methylene Blue. Int. J. Soc. Mater. Eng. Resour..

[33] Ziebro J., Łukasiewicz I., Borowiak-Palen E., Michalkiewicz B. (2010). Low temperature growth of carbon nanotubes from methane catalytic decomposition over nickel supported on a zeolite. Nanotechnology.

[34] Sacaliuc E., Beale A.M., Weckhuysen B.M., Nijhuis T.A. (2007). Propene epoxidation over Au/Ti-SBA-15 catalysts. J. Catal..

[35] Yadav R., Amoli V., Singh J., Tripathi M.K., Bhanja P., Bhaumik A., Sinha A.K. (2018). Plasmonic gold deposited on mesoporous Ti_x_Si_1-x_O_2_ with isolated silica in lattice: An excellent photocatalyst for photocatalytic conversion of CO_2_ into methanol under visible light irradiation. J. CO_2_ Util..

[36] Ravindra A.V., Behera B.C., Padhan P. (2014). Laser Induced Structural Phase Transformation of Cobalt Oxides Nanostructures. J. Nanosci. Nanotechnol..

[37] Xu X., Sun Y., Fan Z., Zhao D., Xiong S., Zhang B., Zhou S., Liu G. (2018). Mechanisms for ·O^−^_2_ and ·OH Production on Flowerlike BiVO_4_ Photocatalysis Based on Electron Spin Resonance. Front. Chem..

[38] Graca I., González L.V., Bacariza M.C., Fernandes A., Henriques C., Lopes J.M., Ribeiro M.F. (2014). CO_2_ hydrogenation into CH_4_ on NiHNaUSY zeolites. Appl. Catal. B Environ..

[39] Lin T.J., Meng X., Shi L. (2014). Ni-exchanged Y-zeolite- An efficient heterogeneous catalyst for acetylene hydrocarboxylation. Appl. Catal. A.

[40] Mohan V., Raghavendra C., Pramod C.V., Raju B.D., Rao K.S.R. (2014). Ni/H-ZSM-5 as a promising catalyst for vapour phase hydrogenation of levulinic acid at atmospheric pressure. RSC Adv..

[41] Deshmane V.G., Owen S.L., Abrokwah R.Y., Kuila D. (2015). Mesoporous nanocrystalline TiO_2_ supported metal (Cu, Co, Ni, Pd, Zn, and Sn) catalysts: Effect of metal-support interactions on steamreforming of methanol. J. Mol. Catal A.

[42] Ivan S.B., Fechete I., Papa F., Marcu I.C. (2021). Ethane oxydehydrogenation over TiP_2_O_7_-supported NiO catalysts. Catal. Today.

[43] Jongsomjit B., Panpranot J., Goodwin J.G. (2001). Co-support compound formation in alumina-supported cobalt catalysts. J. Catal..

[44] Zhang Y., Wei D., Hammache S., Goodwin J.G. (1999). Effect of Water Vapor on the Reduction of Ru-Promoted Co/Al_2_O_3_. J. Catal..

[45] Srisawad N., Chaitree W., Mekasuwandumrong O., Shotipruk A., Jongsomjit B., Panpranot J. (2012). CO_2_ hydrogenation over Co/Al_2_O_3_ catalysts prepared via a solid-state reaction of fine gibbsite and cobalt precursors. React. Kinet. Mech. Catal..

[46] Ji Y., Zhao Z., Duan A., Jiang G., Liu J. (2009). Comparative Study on the Formation and Reduction of Bulk and Al_2_O_3_-Supported Cobalt Oxides by H2-TPR Technique. J. Phys. Chem. C.

[47] Kruatim J., Jantasee S., Jongsomjit B. (2017). Improvement of Cobalt Dispersion on Co/SBA-15 and Co/SBA-16 Catalysts by Ultrasound and Vacuum Treatments during Post-Impregnation Step. Eng. J..

[48] Solsona B., Davies T.E., Garcia T., Vazquez I., Dejoz A., Taylor S.H. (2008). Total oxidation of propane using nanocrystalline cobalt oxide and supported cobalt oxide catalysts. Appl. Catal. B.

[49] Nasir J.A., Islam N., Rehman Z., Butler I.S., Munir A., Nishina Y. (2021). Co and Ni assisted CdS@g-C3N4 nanohybrid: A photocatalytic system for efficient hydrogen, evolution reaction. Mater. Chem. Phys..

[50] Yao S., Tang H., Liu M., Chen L., Jing M., Shen X., Li T., Tan J. (2019). TiO_2_ nanoparticles incorporation in carbon nanofiber as a multi-functional interlayer toward ultralong cycle-life lithium-sulfur batteries. J. Alloys Compd..

[51] Liu L., Li Y. (2014). Understanding the Reaction Mechanism of Photocatalytic Reduction of CO_2_ with H_2_O on TiO_2_-Based Photocatalysts: A Review. Aerosol Air Qual. Res..

[52] Collin F. (2019). Chemical Basis of Reactive Oxygen Species Reactivity and Involvement in Neurodegenerative Diseases. Int. J. Mol. Sci..

